# Optimizing clozapine for chemogenetic neuromodulation of somatosensory cortex

**DOI:** 10.1038/s41598-020-62923-x

**Published:** 2020-04-07

**Authors:** Jongwook Cho, Seungjun Ryu, Sunwoo Lee, Junsoo Kim, Hyoung-Ihl Kim

**Affiliations:** 1 Department of Biomedical Science and Engineering, Gwangju Institute of Science and Technology, Gwangju, Republic of Korea; 2 Department of Neurosurgery, Presbyterian Medical Center, Jeonju, Republic of Korea

**Keywords:** Neuroscience, Neurology

## Abstract

Clozapine (CLZ) has been proposed as an agonist for Designer Receptors Exclusively Activated by Designer Drugs (DREADDs), to replace Clozapine-N-oxide (CNO); however, there are no reliable guidelines for the use of CLZ for chemogenetic neuromodulation. We titrated the optimal dose of CLZ required to evoke changes in neural activity whilst avoiding off-target effects. We also performed [^18^F]Fluoro-deoxy-glucose micro positron emission tomography (FDG-microPET) scans to determine the global effect of CLZ-induced hM3D(Gq) DREADD activation in the rat brain. Our results show that low doses of CLZ (0.1 and 0.01 mg/kg) successfully induced neural responses without off-target effects. CLZ at 1 mg/kg evoked a stronger and longer-lasting neural response but produced off-target effects, observed as changes in locomotor behavior and FDG-microPET imaging. Unexpectedly, FDG-microPET imaging failed to demonstrate an increase in regional glucose metabolism in the stimulated cortex during CLZ chemogenetic neuromodulation. Therefore, caution should be used when interpreting FDG-PET images in the context of cortical chemogenetic activation.

## Introduction

Neuroscientists and neurologists have long dreamed of being able to use non-invasive, target-specific neuromodulators; such tools can be relatively easily translated to treat neurological disorders. Designer Receptors Exclusively Activated by Designer Drugs (DREADDs) consist of engineered muscarinic receptors that respond exclusively to the synthetic ligand Clozapine-N-oxide (CNO). When DREADDs first emerged, they garnered wide attention because of their prolonged action and non-invasive nature^1–3^. Most importantly, the ligand CNO was assumed to be devoid of actions at endogenous neuroreceptors and hence able to modulate specific targets precisely, enabling cell-type specific dissection of neural circuits and behavior. However, recent evidence shows that CNO does not cross the blood−brain barrier and may in fact have low binding affinity for DREADDs^4^. Instead, CNO appears to be converted to clozapine (CLZ) *in vivo*, producing plasma concentrations of CLZ high enough not only to stimulate the DREADDs but also to occupy dopamine and serotonin receptors in the brain^4–6^. Because of this drawback, several alternative chemical compounds, including JHU37152, JHU37160, and compound 21, have been tested in hopes that they may replace the role of CNO^7,8^. However, each of these compounds exhibited a similar target profile but with different off-target or sedative effects. Furthermore, any newly developed compounds would need to pass through FDA screening procedures. Recently, the FDA-approved drug olanzapine has been reported to be a potent activator of the silencing DREADD hM4D(Gi)^9^. However, a ligand that acts effectively at both silencing and activating (e.g. hM3D(Gq)) DREADDs would be most desirable.

CLZ is an atypical antipsychotic drug, most commonly used in patients with treatment-resistant schizophrenia^10^. CLZ has been suggested for use in chemogenetic neuromodulation, in place of CNO, because it readily crosses the blood–brain barrier^4,7^. However, CLZ has some inherent drawbacks. Because CLZ has high uptake in the rat brain, with a brain-to-plasma ratio of 24:1, even low doses of CLZ may induce off-target effects via concurrent action at endogenous CLZ targets^11^. In addition, CLZ is known to influence metabolic activity in multiple areas of the brain^12,13^. Therefore, it is important to find the lowest dose of CLZ that will activate DREADDs while minimizing off-target effects. It is also critical to understand the causal relationship between CLZ and global change of brain activity.

The ability of DREADD-based techniques to modulate neural circuits and behaviors has been tested for a number of brain functions, including appetite, reward, motor behavior, pain, anxiety-like behaviors, etc^14–17^. Thus, several subcortical neural structures or circuits known to be involved in these functions have been used as targets for DREADD-based chemogenetic neuromodulation. In addition, the cortex is an important potential target for chemogenetic neuromodulation because cortical excitability is related with plasticity and motor rehabilitation^18,19^. Recently invasive and noninvasive neuromodulatory techniques, including transcranial magnetic stimulation (TMS), transcranial direct current stimulation (tDCS), and epidural cortical stimulation (ECS), have been used to modulate cortical excitability and induce neuronal plasticity in motor or sensory cortex^20–23^. In particular, neuromodulation of somatosensory cortex may rescue post-stroke deficits in a rodent model of subcortical capsular infarct^24,25^. However, DREADD-driven chemogenetic neuromodulation has not yet been attempted in the cortex.

In the current study, we titrated the optimal dose of CLZ for inducing hM3D(Gq)-driven neuronal stimulation with minimal off-target effects in somatosensory cortex. We further tested the causal relationship between CLZ-chemogenetic neuromodulation (CLZ-ChemoNM) and brain responses by performing behavioral, electrophysiological, and functional imaging (microPET) studies at the systems level to ensure the translatability of CLZ-ChemoNM.

## Results

### CLZ-induced metabolic and behavioral changes in naive rats

**Figure 1:**
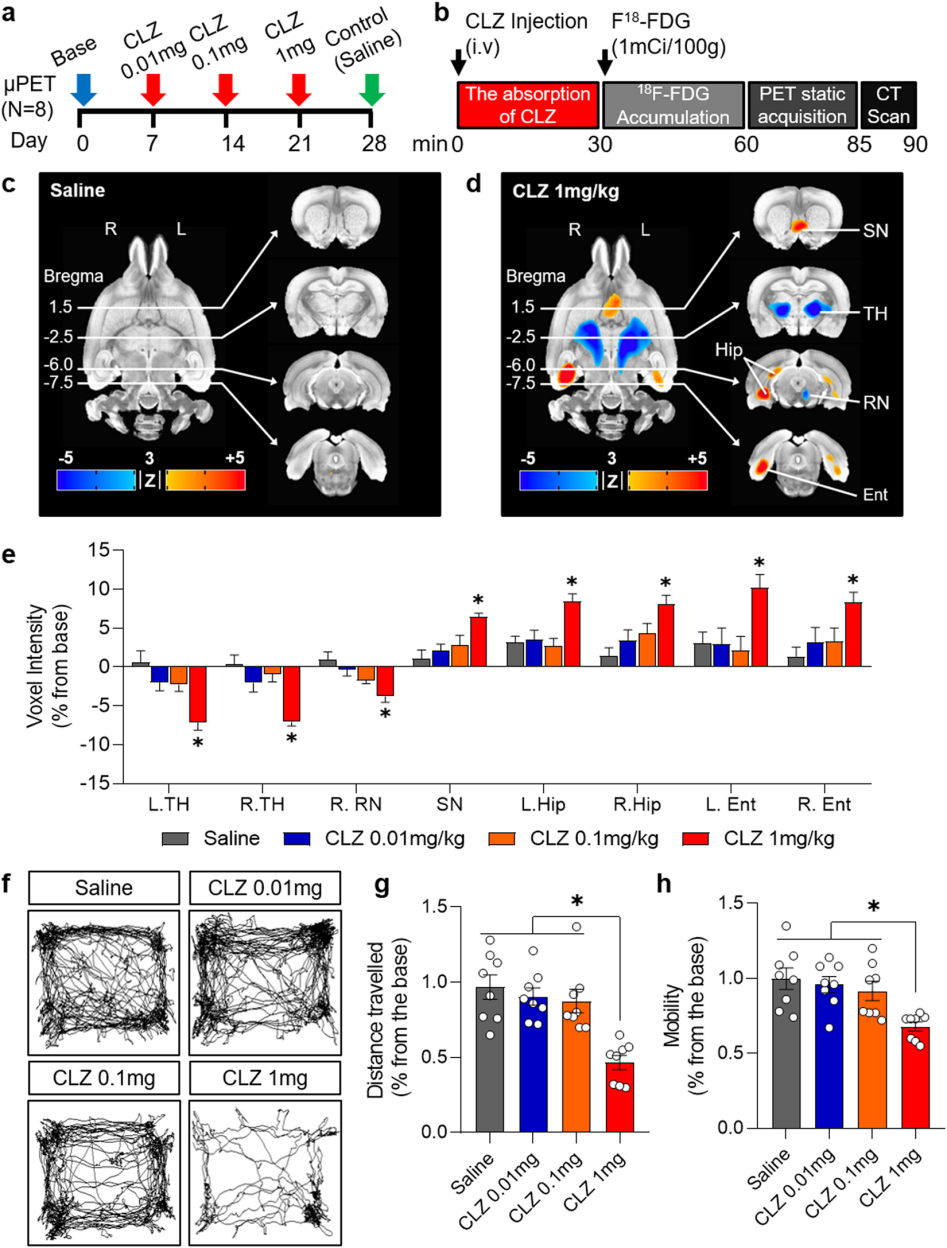
Clozapine-induced metabolic and behavioral changes in naïve rats. (**a**) Experimental design for FDG-microPET scans with different doses of clozapine. (**b**) Timeline of the FDG-microPET scanning procedure. (**c**) FDG-microPET image after administration of saline (N = 8; 3dClustSim, AFNI, α = 0.05, *p* < 0.01, *k* < 39). (**d**) FDG-microPET image after administration of 1 mg/kg clozapine (N = 8; 3dClustSim, AFNI, α = 0.05, *p* < 0.01, *k* < 39). (**e**) Change in voxel intensity for regional glucose metabolism by dose of CLZ and anatomical location (N = 8; compared with the saline group, 1-way ANOVA with Dunn’s multiple comparisons, *p < 0.05). (**f**) Representative traces of locomotor activity in the open field test after administration of CLZ. (**g**) Total distance traveled and (**h**) mobility in the open field test measured over 30 minutes starting 30 minutes after administration of CLZ or saline (N = 8; 1-way ANOVA with Tukey’s multiple comparisons, *p < 0.05). CLZ, clozapine; L, left; R, right; SN, septal nucleus; TH, thalamus; RN, red nucleus; Hip, hippocampus; Ent, entorhinal cortex.

Given that CLZ induces metabolic depression in multiple areas of the brain including the basal ganglia, thalamus, and cortical areas, and consequent changes in behavior^12,26^, we first explored the dose-dependent effects of CLZ in naive rats (i.e., rats not expressing DREADDs) using [^18^F]Fluoro-deoxy-glucose micro positron emission tomography (FDG-microPET) and the open field test. We administered CLZ (Tocris, 0444) at doses of 0.01, 0.1, and 1 mg/kg and saline at 1-week intervals (Fig. 1a). Using an established microPET protocol, animals were injected with CLZ and F^18-^FDG (100 mCi/100 g) through the tail vein during brief isoflurane anesthesia, followed by a 30-minute uptake period. Then, static PET scans (25 minutes) and attenuation-correction computerized tomography (5 minutes) were performed with an Inveon PET/CT scanner^24^ (Siemens Medical Solutions, TN, USA) (Fig. 1b).

Neither administration of CLZ at doses of 0.01 or 0.1 mg/kg nor administration of saline produced significant changes in regional glucose metabolism (rGluM) (Fig. 1c–e; Supplementary Fig. 1). However, administration of 1 mg/kg CLZ produced significant depression of rGluM in the bilateral thalamus and unilateral red nucleus and significant activation of rGluM in the septal nucleus, bilateral ventral striatum, and entorhinal cortices (Fig. 1d,e).

The open field test was performed to determine the behavioral effects associated with the metabolic changes induced by CLZ. 30 minutes after the administration of saline or CLZ at doses of 0.01, 0.1 or 1 mg/kg, the locomotor activity of animals in the open arena was observed. There were no significant differences in distance traveled or mobility between the saline, CLZ 0.01 mg/kg, and CLZ 0.1 mg/kg groups (Fig. 1f–h). However, administration of 1 mg/kg CLZ produced a significant decrease in both distances traveled and mobility (Fig. 1f–h). These data indicate that doses of CLZ of 1 mg/kg or higher induce significant behavioral changes associated with metabolic depression by CLZ, whereas doses of CLZ of 0.1 mg/kg or lower did not induce significant behavioral changes or off-target metabolic effects. Our results suggest that doses of CLZ below 0.1 mg/kg should be used to avoid undesirable nonspecific effects of CLZ during chemogenetic modulation.

### CLZ-ChemoNM evokes neural responses

**Figure 2:**
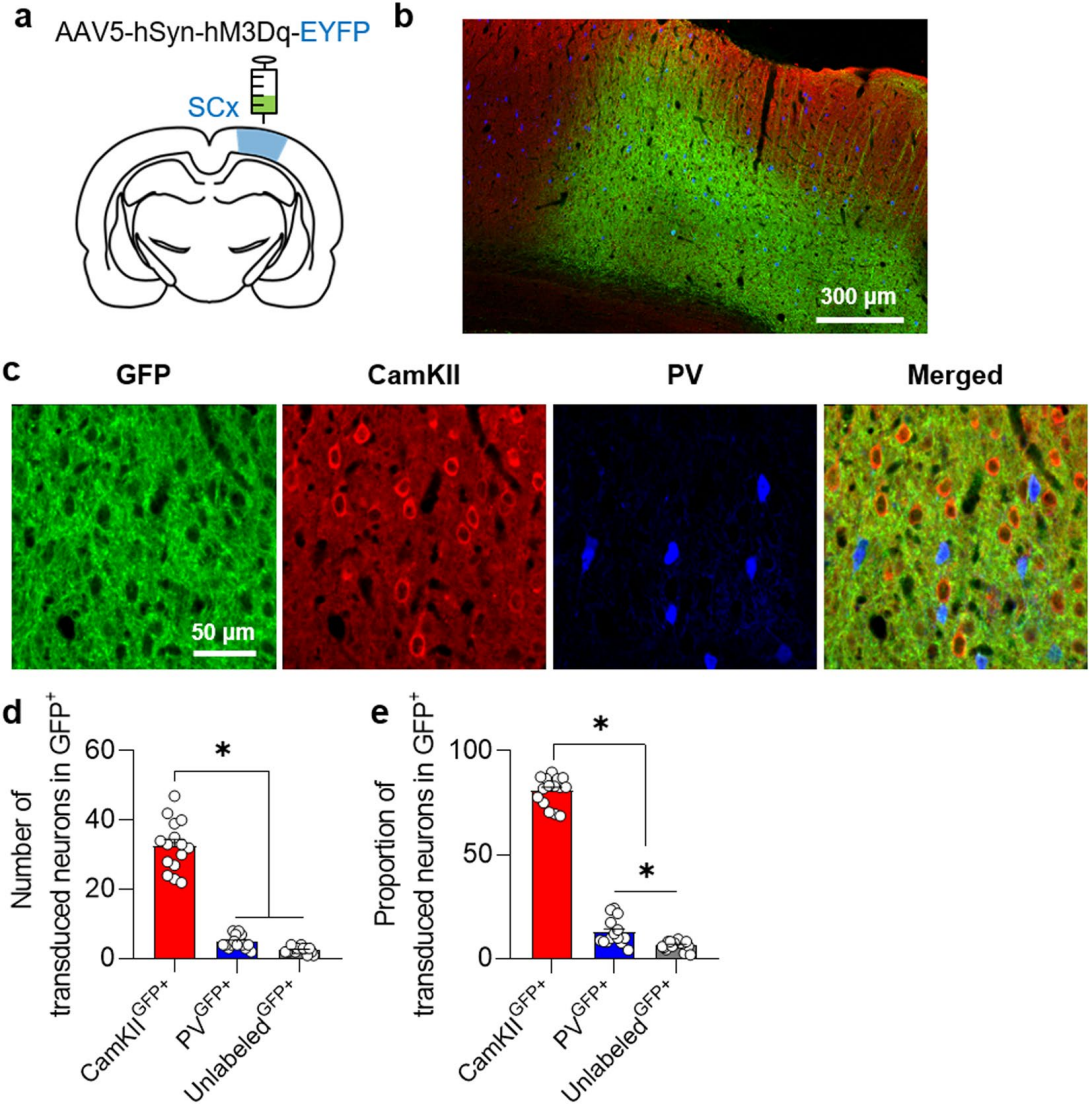
hM3Dq-DREADD viral expression. (**a**) Schematic drawing of DREADD virus delivery. (**b**) Representative confocal images demonstrating virus expression in somatosensory cortex. (**c**) Confocal images showing the co-expression of GFP and cells positive for CamKII and PV. (**d**,**e**), Number and proportion of CamKII, PV, and unlabeled cells in areas expressing virus. (N = 5; 1-way ANOVA with Tukey’s multiple comparisons, *p < 0.05) SC, somatosensory cortex; GFP, green fluorescent protein.

To determine the effect of DREADD-driven excitation of the cortex, we injected the Gq-coupled excitatory DREADD virus AAV5-hSyn-hM3D(Gq)-EYFP (Virus Core Facility, KIST, Seoul, Korea) into the somatosensory cortex of rats (Fig. 2a–c). After waiting approximately 3 weeks for viral expression, we quantified the number of CamKII and PV positive cells in the hM3Dq-expressing area (Fig. 2d,e). The percentages of CamKII and PV positive cells were 80.8 ± 6.89% and 12.75 ± 6.2%, respectively. We also found cells that were unlabeled by either of these two antibodies (6.43 ± 2.16%).

**Figure 3:**
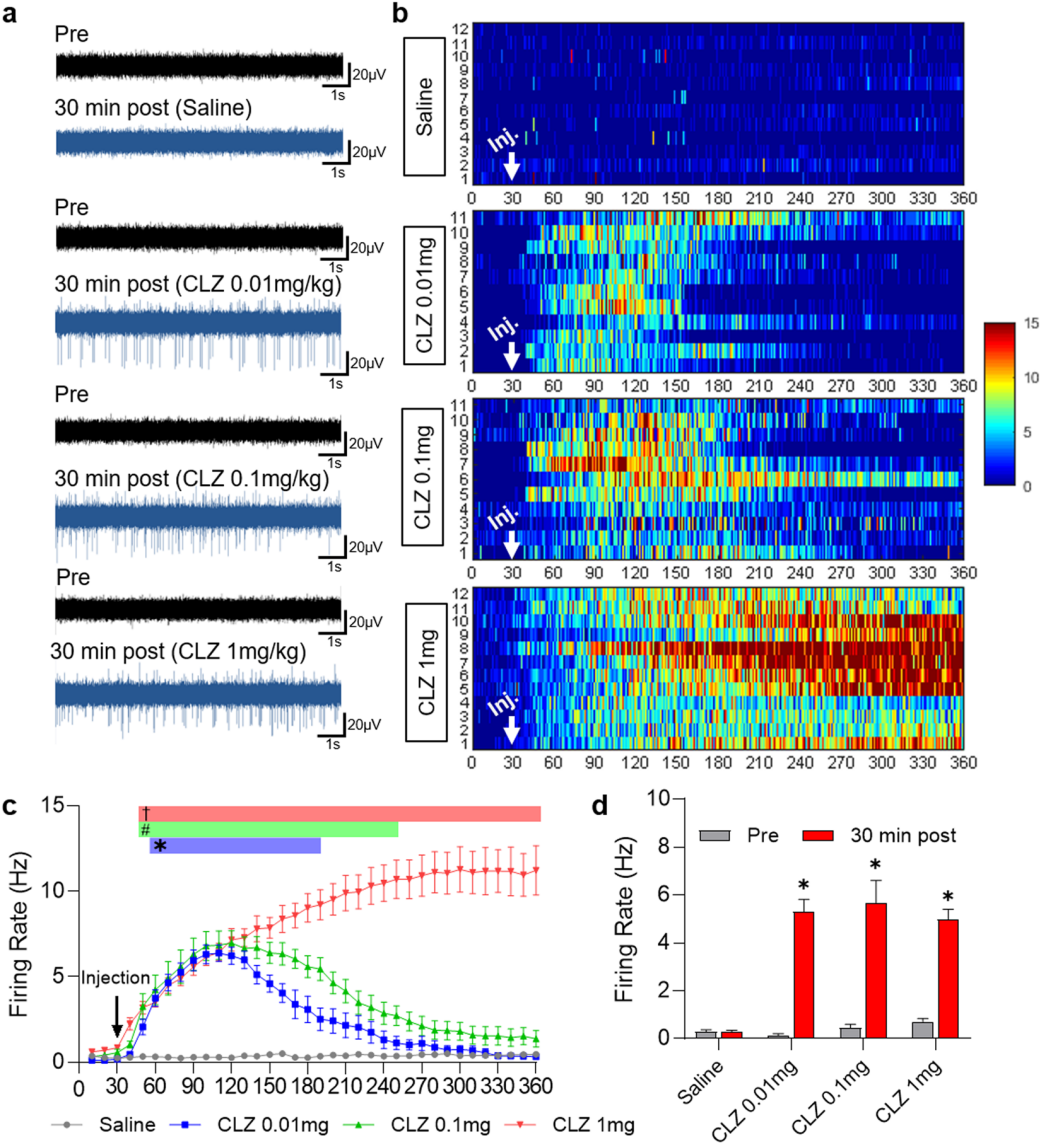
Neural response in the brain after CLZ chemogenetic neuromodulation. (**a**) Representative samples of extracellular recording before (pre) and 30 min. after (post) administration of saline or different doses of CLZ. (**b**) 2-D raster plot showing that spiking activity over 360 min. depends on the dose of CLZ. Numbers on the Y axis indicate the number of recordings. Scale bar at the right indicates the frequency of neuronal firing (Hz). (Saline (N = 5, n = 12), CLZ 0.01 mg/kg (N = 4, n = 12) CLZ 0.1 mg/kg (N = 5, n = 11) CLZ 1 mg/kg (N = 5, n = 12)) (**c**) Time-dependent distribution of the average firing rate after administration of saline or different doses of CLZ (2-way ANOVA with Dunn’s multiple comparisons, *p* < 0.05. *CLZ 0.01 mg/kg vs. saline; ^#^CLZ 0.1 mg/kg vs. saline; ^†^CLZ 1 mg/kg vs. saline). (**d**) Comparison of firing rates between before and 30 min after CLZ administration. (Student’s t-test, * *p* < 0.05).

To confirm that CLZ-ChemoNM induced neuronal depolarization via hM3Dq^27^, we performed electrophysiological studies (Fig. 3a–d). First, we inserted a 32 channel silicone probe^28^ into the area previously injected with virus, before administering different doses of CLZ and saline. Administration of saline did not alter the firing rate. Administration of 0.01 mg/kg CLZ produced an increase in firing 22 minutes after CLZ injection, and the increase persisted for 145 minutes. Administration of 0.1 mg/kg CLZ produced an increase in firing 16 minutes after CLZ injection, and this increase persisted for 209 minutes. Similarly, administration of 1 mg/kg CLZ produced an increase in firing 14 minutes after CLZ injection, which persisted markedly longer than 6 hours. Firing rates were quantified by averaging over 30-minute blocks of pre-injection and post-injection 30 minutes after injection (Fig. 3d). Taken together, these results indicate that administration of CLZ can induce the chemogenetic activation. The higher the administered dose of CLZ, the greater and longer was the extent of the neural depolarization

**Figure 4:**
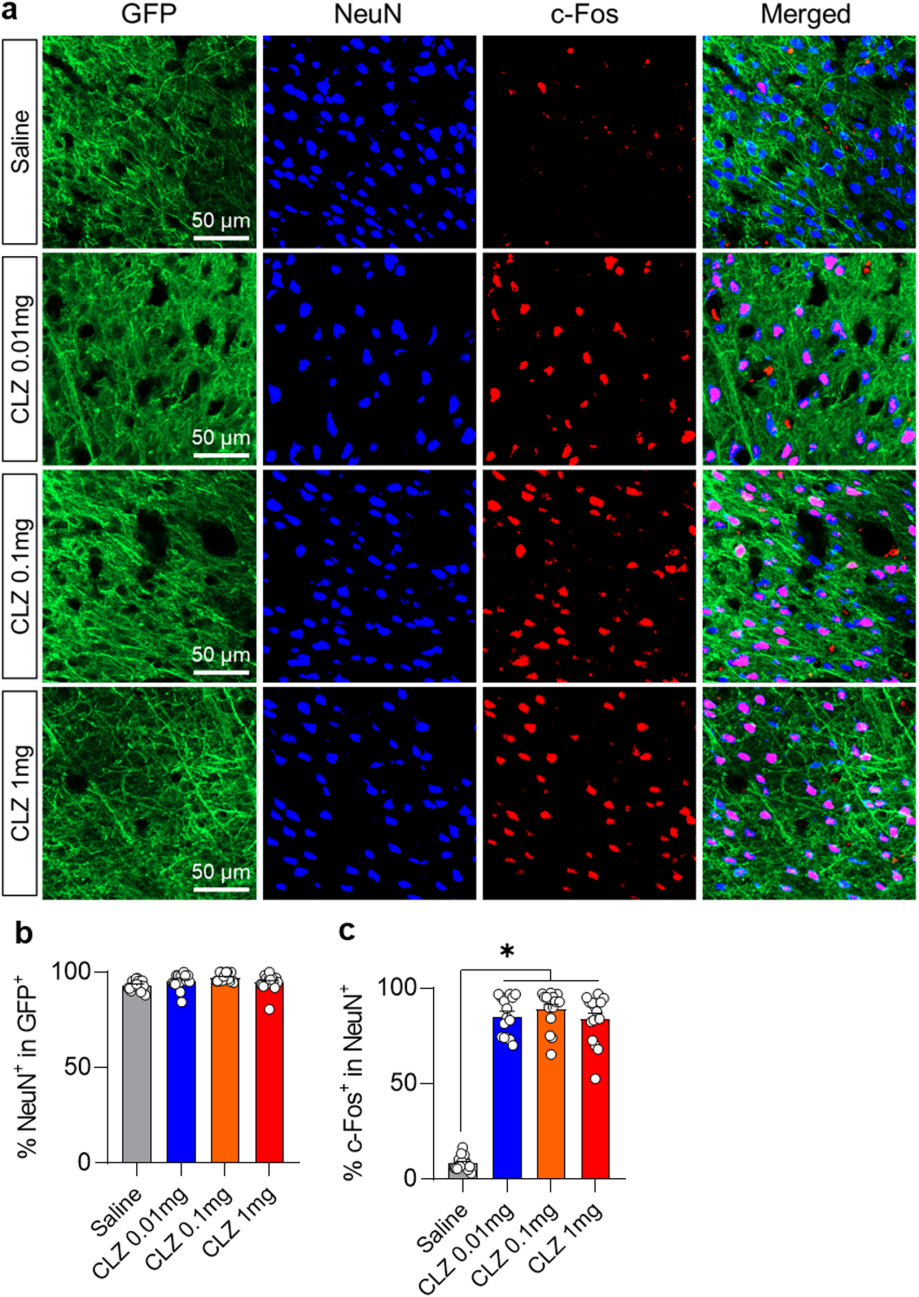
c-Fos expression after administration of different doses of CLZ. (**a**) Representative sections from the somatosensory cortices showing c-Fos expression after different doses of CLZ. (**b**) Quantitative comparison of cells co-labeled for GFP and NeuN after different doses of CLZ. (**c**) Quantitative comparison of cells co-labeled for c-Fos and NeuN after different doses of CLZ. (Saline (N = 5), CLZ 0.01 mg/kg (N = 5), CLZ 0.1 mg/kg (N = 5), CLZ 1 mg/kg (N = 5); 1-way ANOVA with Tukey’s multiple comparisons, *p < 0.05).

CLZ administration also produced a significant increase in c-Fos expression compared with the saline administration group (Fig. 4a–c). The percentage of cells co-labeled for GFP and NeuN did not differ significantly among the groups (Fig. 4b), however, there was significantly more co-labeling of NeuN and C-Fos in the CLZ administration groups than in the saline administration group. The percentage of cells co-labeled by c-Fos staining and NeuN was 8.5 ± 3.61%, for the saline group, 85.25 ± 10.12% for the 0.01 mg/kg CLZ group, 89.08 ± 9.93% for the 0.1 mg/kg CLZ group, and 83.67 ± 12.63% for the 1 mg/kg CLZ group (Fig. 4c).

### FDG-microPET shows the off-target effects of CLZ but fails to reflect the chemogenetic cortical stimulation

**Figure 5:**
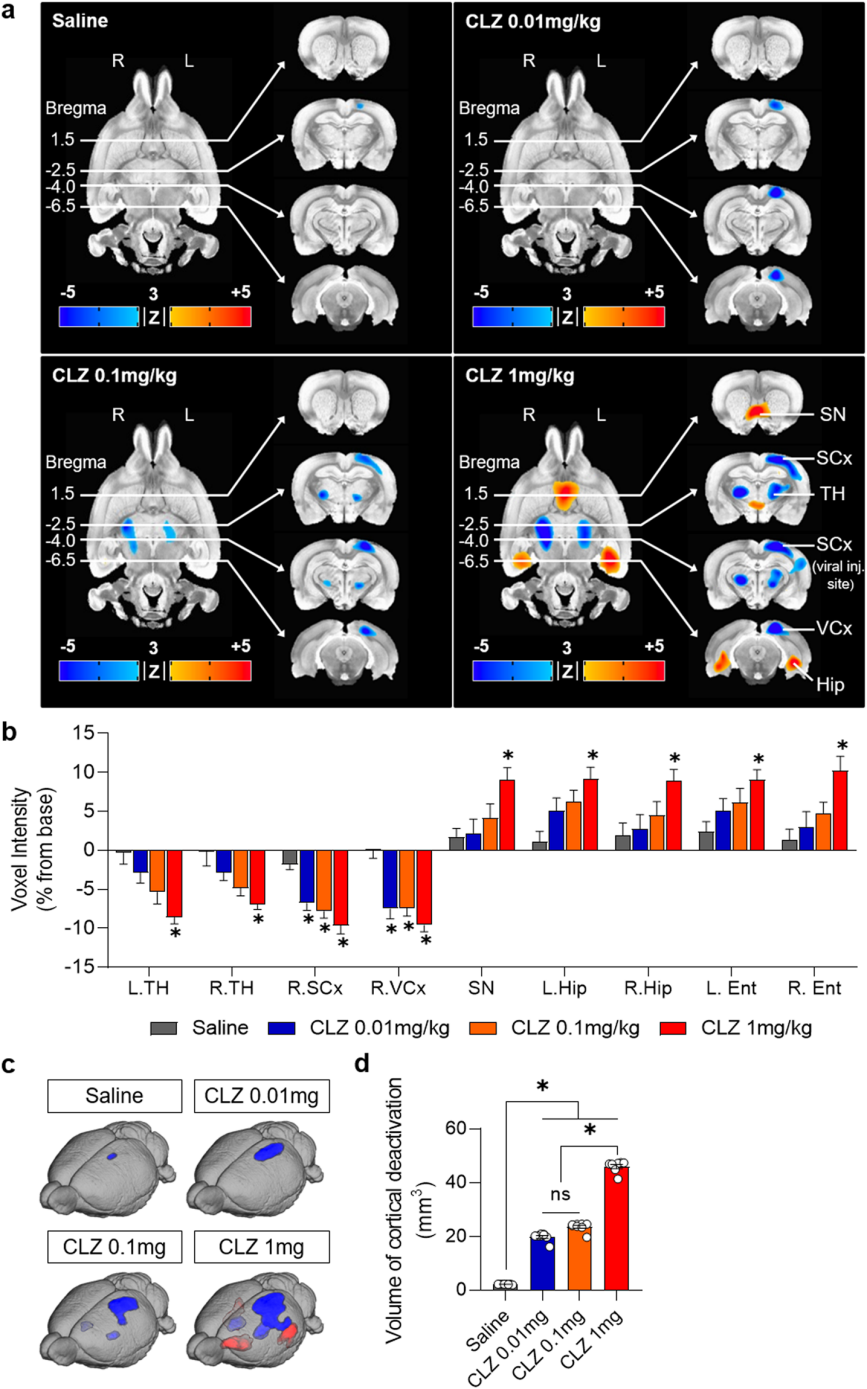
FDG-microPET imaging after CLZ chemogenetic neuromodulation. (**a**) FDG-microPET images after saline or CLZ-ChemoNM with different doses of CLZ (N = 8; 3dClustSim, AFNI, α = 0.05, *p* < 0.01, *k* < 39). (**b**) Voxel intensity (% change from the baseline) for regional glucose metabolism after CLZ-ChemoNM by dose of CLZ and anatomical location (*N* = 8; 1-way ANOVA with Dunn’s multiple comparisons, *p < 0.05 compared with the saline group) (**c**) FDG-microPET images of cortical deactivation after CLZ-ChemoNM with saline and different doses of CLZ. (**d**) Volume of cortical deactivation after CLZ-ChemoNM (N = 8; 1-way ANOVA with Tukey’s multiple comparisons, *p < 0.05). CLZ, clozapine; CLZ-ChemoNM, CLZ chemogenetic neuromodulation; L, left; R, right; SN, septal nucleus; SCx, somatosensory cortex; VCx, visual cortex, TH, thalamus; Hip, hippocampus.

Next, we wanted to examine whether the local chemogenetically induced changes in excitability are reflected in changes in rGluM. Given that CLZ itself depresses brain metabolism in selected areas of the brain, we hypothesized that CLZ-ChemoNM may show complicated pattern of brain activation in somatosensory cortex. To examine this in detail, we analyzed changes in rGluM with CLZ or saline in animals injected with AAV5-hSyn-hM3D(Gq)-EYFP (CLZ-ChemoNM). In both naive and virus-expressed rats, administration of saline did not produce any significant changes in rGluM in somatosensory cortex (Figs. 1c and 5a) while administration of CLZ, regardless of dose, resulted in metabolic depression in the somatosensory cortex for voxel intensity and standard uptake value (SUV) for regional glucose metabolism (Fig. 5a,b; Supplementary Fig. 2). Furthermore, the area of cortical depression in somatosensory cortex increased as the dose of CLZ increased (Fig. 5c,d). These results are contrary to expectations: virus-expressing regions would be expected to show an increase in rGluM with CLZ-ChemoNM.

Administration of CLZ (0.1 mg/kg) produced metabolic depression in the bilateral thalami (Fig. 5a,b). Administration of CLZ at a higher dose (1 mg/kg) showed metabolic depression in the bilateral thalami and unilateral red nucleus but an increase in rGluM in the septal area, bilateral hippocampus, and entorhinal cortices (Fig. 5a,b), similar to the off-target effects in naive rats shown in Fig. 1d.

Taken together, these results suggest that CLZ produces similar off-target effects in both naive and CLZ-ChemoNM rats. CLZ-driven cortical activation of the somatosensory cortex was not reflected in the FDG-microPET scans. Rather, we observed cortical depression at the site of virus injection. Thus caution should be exercised when assessing the effects of chemogenetic cortical stimulation using FDG-microPET.

The open field test was used to determine the behavioral effects of CLZ-driven DREADD activation (Supplementary Fig. 3). After administration of saline or CLZ (0.01, 0.1, or 1 mg/kg), locomotor activity in the open arena was measured. As in the naive rats, CLZ-ChemoNM rats showed no significant differences in mobility or distance traveled between the saline CLZ 0.01 mg/kg, and CLZ 0.1 mg/kg groups, but there was a decrease in both with the 1 mg/kg CLZ dose. These data indicate that CLZ induces a similar dose-dependent behavioral response in both naive and CLZ-ChemoNM animals. Targeting the somatosensory cortex with acute chemogenetic activation did not produce changes in open field behavior above and beyond those observed with CLZ in naive animals.

## Discussion

In this study, we evaluated the effects of CLZ-ChemoNM via the hM3D(Gq) DREADD on locomotor activity, spike firing, and FDG-PET imaging to validate the usefulness of this tool for future ongoing experiments. As CLZ produces metabolic changes in multiple areas of the brain, this situation requires careful testing to determine the appropriate CLZ dosage and to interpret the behavioral and imaging results.

The use of CNO to activate DREADDS has become controversial since the discovery that CNO does not cross the blood–brain barrier and that activation of DREADDs *in vivo* is likely mediated by the conversion of CNO to CLZ^4,6,29^. Thus subthreshold 0.1 mg/kg dose of CLZ have been suggested as an alternative activator for DREADDs^4^. Other report suggested that a CLZ dose between 0.2 and 0.5 mg/kg should be used in experiments involving hM4Di and that a dose between 0.5 and 1 mg/kg should be used in experiments with hM3Dq^29^. In this study, we showed that CLZ at ‘much lower doses’ (0.01 and 0.1 mg/kg) induced hM3Dq activation without off-target effects. However, 1 mg/kg of CLZ had off-target effects, as revealed by FDG-microPET imaging, whilst having the strongest and longest lasting chemogenetic effect, as shown by increases in cell firing. We therefore suggest that low doses of CLZ, such as 0.01 or 0.1 mg/kg, are suitable for activating DREADDs without off-target effects. The dose selected for any particular experiment will ultimately depend on the intensity and duration of chemo-stimulation required in the experiment.

In our FDG-microPET study to determine the off-target effects of CLZ, administration of 1 mg/kg of CLZ in naive rats produced significant depression of rGluM in the somatosensory cortex, bilateral thalamus, and unilateral red nucleus but significant activation of rGluM in the septal nucleus, bilateral ventral striatum, and hippocampi. In rats expressing DREADDs, somatosensory cortex (where the virus was expressed) showed additional metabolic depression with increasing doses of CLZ. These changes in rGluM are different from those reported in human studies, which showed decreased prefrontal and basal ganglia rGluM with CLZ but increased rGluM in visual areas^26^. However, preclinical data have shown decreases in rGluM in thalamic, cortical, and limbic areas after acute and chronic CLZ treatment in rats^12^. These finding are consistent with our FDG-microPET findings. Therefore, we believe that FDG-PET imaging can provide reliable information regarding the off-target effects of CLZ and can be used to help determine the optimal dose of CLZ for CLZ-ChemoNM.

Previous PET studies have assessed the brain penetration, affinity, and *in-vivo* DREADD occupancy of potential DREADD agonists using a PET DREADD radiotracer^4,30^, and CNO-driven DREADD effects on rGluM have been reported to alter FDG uptake in the multiple areas of the brain^26,31–33^. Generally, FDG-microPET imaging can indicate the status of neural activity coupled to increases or decreases in rGluM during neuromodulation^34^. However, we did not observe any increases in metabolic activity in somatosensory cortex with FDG-microPET imaging in this study, despite observing electrophysiological activation of DREADDs by CLZ in this area. We hypothesize that the metabolic deactivation we observed may be produced by special conditions related to the chemogenetic activation induced by CLZ: although GPCR signaling induced by DREADD stimulation evokes complex signaling cascades, interactions with various channels, and phosphorylation of diverse proteins^35^, the energy consumption of GPCR signaling is much lower than that of synaptic ion channels, which are the major consumer of energy in the brain^36^. Thus it is possible that this underlies the activation pattern observed with microPET imaging. Further, there are several confounding factors that may depress metabolic activity during chemogenetic activation by CLZ. Given that microPET images are the result of the subtraction of positive and negative factors ^34,37^, the images in this study result from interactions between the low-metabolic GPCR signaling and several confounding factors. These confounding factors include metabolic depression in somatosensory cortex caused by CLZ directly^26,33^, decreased thalamic output to the somatosensory cortex due to CLZ-induced thalamic depression^38^, the influence of CLZ on diffuse projection systems such as dopamine, norepinephrine, and serotonin, and injury to the somatosensory cortex caused by the needle used to inject the virus induction^34^.

This study has some limitations. Animals were not under anesthesia during the waiting period between injection of FDG and scanning; thus we cannot isolate the effects of direct hM3Dq activation from the gross effects of chemogenetically induced changes in behavior. This limits the interpretation of the FDG-PET results in our study. It is possible that we may have observed the increase in FDG uptake, as reported previously^32^ and as we observed in electrophysiology experiments, if the animals had been anesthetized during FDG uptake. However, anesthesia is known to produce variability in glucose levels and tracer uptake, relative to the awake condition^39,40^. In particular, isoflurane anesthesia reduces glucose uptake in the cortex, thalamus, basal ganglia, etc., regions in which we wished to study off-target effects^41^. Because of these considerations, we opted to perform DREADD activation without anesthesia; however, further studies are required to delineate the effects of anesthesia on brain activation under chemogenetic activation.

Although CLZ can interact with genetic factors to evoke rare but potentially fatal complications such as agranulocytosis and neutropenia^42^, CLZ is currently one of the most commonly used atypical antipsychotic drugs for treatment-resistant schizophrenia^10^. As CLZ readily crosses the blood–brain barrier, it can be used reliably to activate DREADD receptors in a variety of chemogenetic experiments, replacing CNO. Further, a sufficiently low dose of CLZ decreases the occurrence of complications and off-target effects. However, it will be necessary to gain a full understanding of how CLZ-induced metabolic depression influences the effects of CLZ-ChemoNM.

## Methods

### Experimental animals

Experiments were performed on male Sprague-Dawley rats (N = 71; 9 weeks old; 300–400 g). All experiments and procedures were approved by Gwangju Institute of Science and Technology Animal Care and Use Committee. Animal ARRIVE guideline were followed in the preparation of the manuscript. Animals were housed two per cage in a room with controlled temperature (21 °C) and humidity (50%) under a 12 h light/dark cycle (07:00–19:00) and had free access to food and water.

For the investigation into the effects of CLZ on rGluM and behavior, 16 wild-type rats were used (FDG-microPET experiment: N = 8; behavioral testing: N = 8). Thirty-five rats underwent AAV5-hSyn-hM3D(Gq)-EYFP (Virus core facility, KIST, Seoul, Korea) injection into somatosensory cortex for DREADD virus expression. These animals were randomly allocated to groups for FDG-PET (N = 8), behavioral testing (N = 8), or electrophysiological validation (N = 19). In addition, 20 rats were injected with the same virus and sacrificed for c-Fos immunohistochemistry (N = 5 for each of the saline and 0.01, 0.1, and 1 mg/kg CLZ groups).

### Viral vector injection

Rats were anaesthetized with a mixture of ketamine hydrochloride (100 mg/kg) and xylazine (7 mg/kg) via intramuscular injection and placed into an animal stereotaxic frame. Body temperature was maintained at 37 ± 0.5 °C. After making a midline scalp incision, a small hole was drilled for viral injection into somatosensory cortex (coordinates from bregma: AP = − 4.0 mm; ML = 3 mm; DV = − 1 mm). One microliter of AAV5-hSyn-hM3D(Gq)-EYFP was stereotactically injected into the target region at a rate of 0.1 ml/min using a micropump (WPI, Sarasota, FL, USA). After the injection, we waited 10 min before slowly retracting the needle and then secured the scalp wound. The animal was then released and transferred to a recovery chamber with pain control (ketoprofen, 2 mg/kg, i.m.). DREADD-related experiments were performed after waiting 3 weeks for viral expression.

### MicroPET Image acquisition and processing

We performed FDG-PET scans to investigate changes in rGluM after CLZ administration in wild-type animals and animals injected with DREADD virus. Each rat underwent five scanning sessions within one week: a baseline scan, scans with injections of 0.01, 0.1, or 1 mg/kg CLZ, and a control scan with a saline injection (Fig. 1a).

Before scanning, animals were fasted for 12 hours to attain consistency in blood glucose levels. During FDG-PET scanning, vital signs were monitored and recorded (BioVet, m2m Imaging Corp., Cleveland, OH, USA), including body temperature (37.0 ± 1 °C), respiration (50 ± 5 respirations per minute), and heart rate (280 ± 20 beats/min).

The rats were injected with CLZ or saline (i.v.) during brief isoflurane anesthesia (1.5%). Thirty minutes after CLZ or saline injection, the rats were injected with ^18^F-FDG (0.1 mCi/100 g) through the tail vein. After a 30-minute uptake period, rats were anesthetized with 2% isoflurane and placed in the microPET scanner under head fixation. We then performed a 25-minute static PET scan and a 5-minute attenuation-correction computed tomography (CT) scan^24^. After completing the microPET/CT scans, the acquired images were reconstructed with a 3-D OSEM/MAP iterative algorithm. Image analysis was performed with the MINC tool kit (McConnell Brain Imaging Centre, Montreal Neurological Institute, Montreal, Canada) and the AFNI packages^43^ (National Institutes of Health, MD, USA). All acquired images were co-registered to an MRI template of the Sprague Dawley rat brain^44^. Images were normalized to the mean value of voxel intensity for the whole brain and spatially smoothed using a 3-D isotropic Gaussian kernel with 1.2 mm full width at half maximum. The 3-D rendered images were acquired using the MRIcroGL (http://www.cabiatl.com/mricrogl/).

### Behavioral testing

The open field test was used to measure changes in locomotor activity after CLZ-ChemoNM^4,45^. Rats were habituated to the open field chamber (50 × 50 × 45 cm) for 30 minutes once before the start of the experiments. Then, rats underwent five testing sessions over 5 weeks. During the testing sessions, animals were placed in the chamber 30 minutes after being injected (i.p.) with vehicle, CLZ (0.01 mg/kg, 0.1 mg/kg, 1 mg/kg) or saline and locomotor activity was recorded for 30 minutes using a video camera placed above the chamber. We quantified mobility and the total distance traveled using open-source analysis software (Bonsai, http://www.open-ephys.org/bonsai).

### Electrophysiological recording

We used electrophysiology to assess changes in spike firing induced by CLZ-ChemoNM in rats expressing DREADD virus. Animal were head fixed in a stereotactic frame under urethane anesthesia (1.5 g/kg, i.p.) and body temperature was maintained at 37 °C with an automatic heating pad. We made a small craniotomy over the site of viral expression (from bregma: AP = − 4.0 mm; ML = 3 mm; DV = − 1 mm). After removing the dura, the exposed area was covered with mineral oil to prevent drying. A 32-channel silicone microprobe^28^ was introduced slowly into the cortex and the basal rhythm was recorded for 30 min. Each rat received just a single dose of CLZ; different rats were used to test the effects of different doses.

Raw data were band-pass filtered (100 Hz – 8 kHz), amplified (20 × 1000), and digitized and stored at a 40 kHz sampling rate for offline analysis with an OmniPlex acquisition system (Plexon, Dallas, TX). Spike analysis was performed using spike sorting software (Offline Sorter, Plexon, Dallas, TX, and NeuroExplorer, Nex Technologies, Littleton, MA, USA). The data were binned on 1-minute interval for calculating the duration of DREADD’s effect. In addition, the average firing rates before and after CLZ injection were calculated as the average frequency of spike discharge during the 30 min period before CLZ injection and during the 30 min period after CLZ injection. If the spike frequency in the period after CLZ injection was more than 20% higher than the basal (pre-injection) firing rate, the increase in firing was considered significant^46^. After recordings were completed, the rats were sacrificed and the brain was extracted and kept in 4% paraformaldehyde (PFA) overnight. Brains were then sliced at 40-µm thickness to confirm the viral expression and electrode tract.

### Immunohistochemeistry

Twenty rats injected with DREADD virus in the somatosensory cortex were sacrificed and processed for c-Fos immunohistochemistry. CLZ [0.01 mg/kg (N = 5), 0.1 mg/kg (N = 5), 1 mg/kg (N = 5)] or saline (N = 5) was intraperitoneally injected. After 150 min, rats were anesthetized with ketamine (100 mg/kg body weight) and perfused with 4% PFA solution. After post-fixing overnight in 4% PFA and cryoprotecting in 30% sucrose in PBS for 3 days, the brains were serially cut with a 40-µm thickness at 200-µm intervals. To confirm expression of DREADDs, GFP staining (1:1000, A11122, Molecular Probes) was performed. Additionally, anti-parvalbumin staining (1:1000, 195 004, Synaptic Systems) for inhibitory neurons and anti-CamKII staining (1:200, AB22609, Abcam) for pyramidal neurons were performed to measure the proportion of transduced cells. In each animal, we selected three ROIs (200 μm × 200 μm) from the hM3Dq-expressing area within which to count cell numbers. Similar immunohistological procedures were performed for GFP to confirm viral expression in the rats that completed FDG-PET scans. Anti c-Fos (1:1000, 2250 S, Cell Signaling) and anti-NeuN (1:200, MAB377, Chemicon) were used to confirm DREADD activation in the target areas. The numbers of c-Fos and NeuN positive cells were counted using the same method.

### Statistical analysis

Data were analyzed with statistical analysis software (Prism, V 7.0; GraphPad, San Diego, CA, USA). The behavioral data and c-Fos activation data were analyzed with one-way analyses of variance with Tukey’s post-hoc comparison (p < 0.05). The time-dependent distribution of average firing rate was analyzed with a two-way analysis of variance with Dunn’s multiple comparison (p < 0.05). A Student’s t-test was used to compare the firing rates in the 30-minute pre-injection and post-injection periods (p < 0.05).

To identify significant changes in rGluM after CLZ injections in control and CLZ-chemoNM animals, a group-level ANOVA was performed using the 3dANOVA in AFNI to compare pre-injection versus post-injection scans (CLZ 0.01 mg/kg, 0.1 mg/kg, 1 mg/kg, and saline). The statistical maps were overlaid on a template to show regions of the brain with significant metabolic changes. The results were corrected for multiple comparisons (3dClustSim, AFNI, α = 0.05, *p* < 0.01, k < 39). ROIs were defined manually in the activated regions for each group. Voxel intensities in each ROI were analyzed with a one-way analysis of variance with Dunn’s multiple comparison (*p* < 0.05). The volume of cortical deactivation was analyzed using a one-way analysis of variance with Tukey’s post-hoc comparison (p < 0.05).

## Supplementary information

Supplementary information.
