# Supplementary material for: Optimizing clozapine for chemogenetic neuromodulation of somatosensory cortex
Source: Sci Rep. 2020 Apr 7;10:6001. doi: 10.1038/s41598-020-62923-x (PMC7138833; doi:10.1038/s41598-020-62923-x)
Supplement: Supplementary file 1 — Supplementary information. [file 41598_2020_62923_MOESM1_ESM.pdf]

Supplementary Material for

# Optimizing clozapine for chemogenetic neuromodulation of somatosensory cortex

Jongwook Cho[1†], Seungjun Ryu[1†], Sunwoo Lee[1], Junsoo Kim[1], Hyoung-Ihl Kim[1,2*]

[1]Department of Biomedical Science and Engineering, Gwangju Institute of Science and Technology, Gwangju, Republic of Korea.

[2]Department of Neurosurgery, Presbyterian Medical Center, Jeonju, Republic of Korea.

[*]Corresponding author. Email : hyoungihl@gist.ac.kr

**This PDF file includes:**

Supplementary Figs. S1 to S3

Supplementary Figure 1. FDG-microPET image after administration of saline and different dose of CLZ.

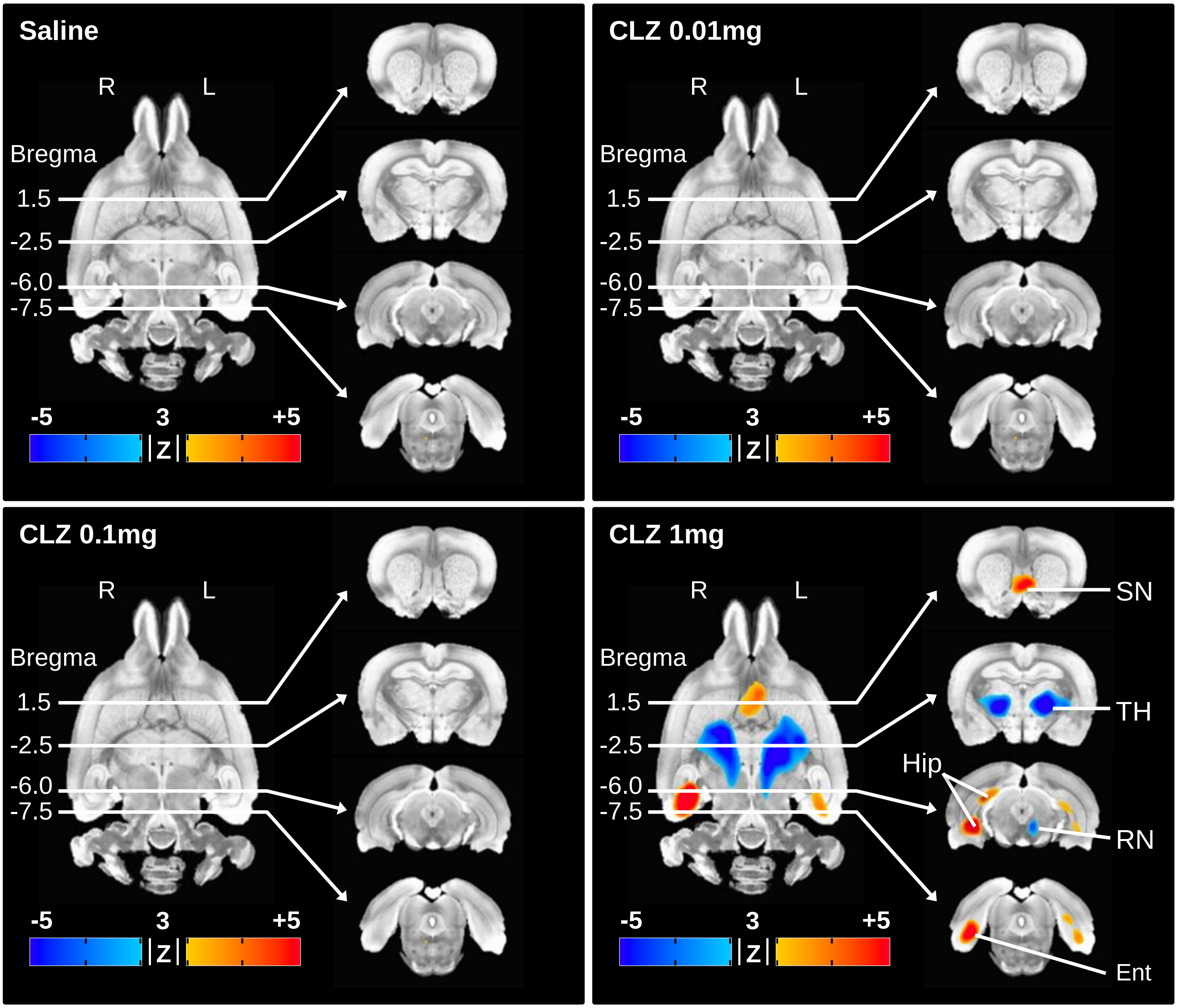


FDG-microPET image after different dose of CLZ and saline in wild rat. (N = 8; 3dClustSim, AFNI, $\alpha = 0.05$, $p < 0.01$, $k < 39$). CLZ, clozapine; L, left; R, right; SN, septal nucleus; TH, thalamus; RN, red nucleus; Hip, hippocampus; Ent, entorhinal cortex

Supplementary Figure 2. Comparison of SUV in somatosensory cortex with different dose of CLZ –induced chemogenetic neuromodulation.

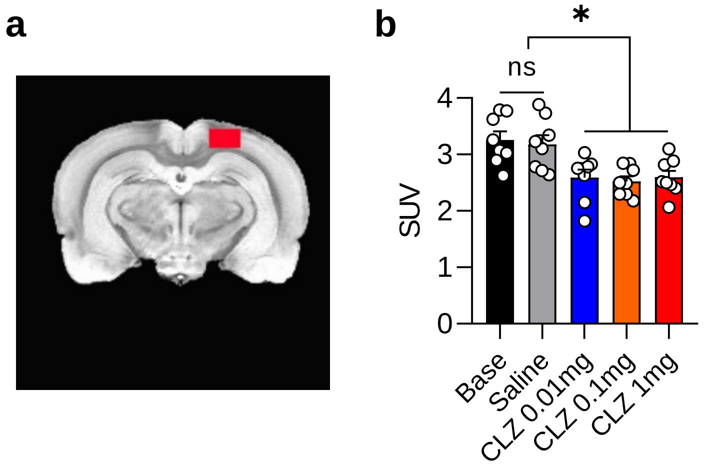


(a) Coronal image of a rat brain indicating the region of interest for SUV analysis (red box). (b) SUV for regional glucose metabolism in somatosensory cortex after CLZ-ChemoNM. (N = 8; 1-way ANOVA with Tukey`s multiple comparisons, *$p < 0.05$).

Supplementary Figure 3. Locomotor behavioral changes after CLZ-ChemoNM

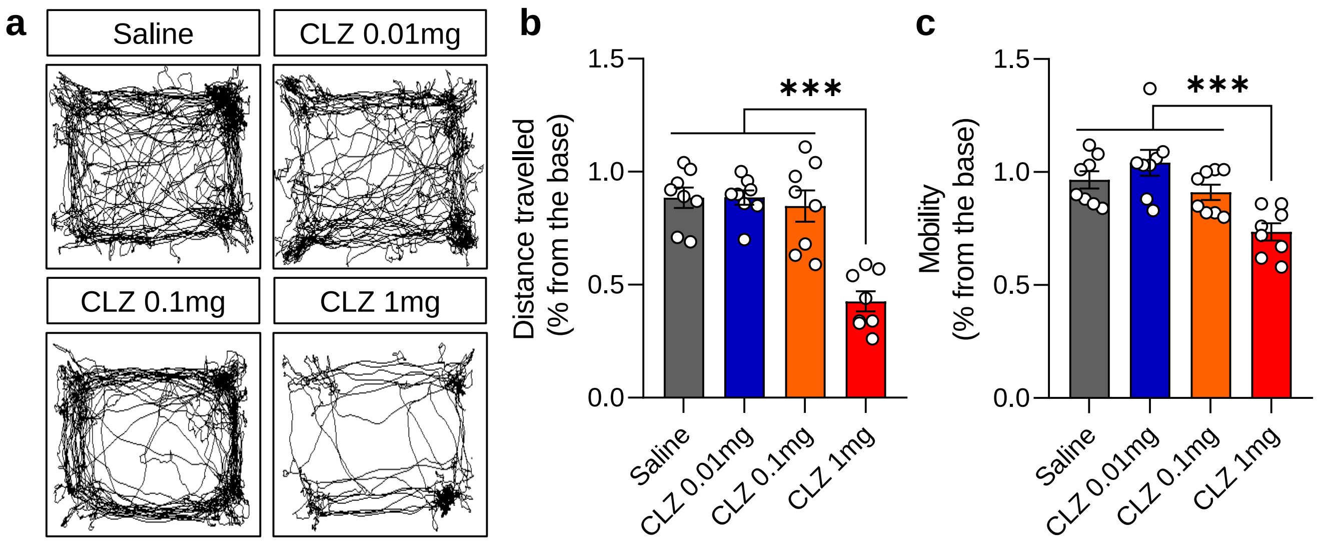


(a) Representative traces of locomotor activity in open field test after CLZ-ChemoNM (b) Total distance travel and (c) mobility 30 minute after administration of CLZ or saline in open field test during 30 minutes (N = 8; 1-way ANOVA with Tukey`s multiple comparisons, *$p$ < 0.05).
